# Internal Microbiota Guided Stage Selection in Two Swine-Manure Bioconversion Flies for Feed-Protein Harvest

**DOI:** 10.3390/insects17040353

**Published:** 2026-03-24

**Authors:** Huiming Zhong, Siyao Wang, Zhen Li, Miao Hong, Dekai Zhang, Zhiyuan Ma, Guanjie Yan

**Affiliations:** Henan Key Laboratory of Insect Biology, Henan Province Engineering Research Center of Insect Bioreactor, China-UK International Joint Laboratory for Insect Biology of Henan Province, Nanyang Normal University, 1638 Wolong Road, Nanyang 473061, China; 19561901371@163.com (H.Z.); 15656361215@163.com (S.W.); lizhenmail1118@163.com (Z.L.); 15237623759@163.com (M.H.); 17538952885@163.com (D.Z.); 17639110525@163.com (Z.M.)

**Keywords:** Diptera, Calliphoridae, Sarcophagidae, biosafety risk, 16S rRNA

## Abstract

Coprophagous flies can convert livestock manure into protein-rich larval biomass for animal feed, but manure-based rearing raises biosafety concerns. This study characterized the internal bacterial community dynamics across development in *Aldrichina grahami* and *Boettcherisca peregrina* reared on swine manure, aiming to identify developmental stages with a lower microbial hazard profile. Using 16S rRNA gene amplicon sequencing of pooled internal samples, we analyzed communities from third-instar larvae, dispersing-stage larvae, pupae at multiple time points, and newly emerged adults. Developmental stage strongly structured bacterial composition and altered richness in both species. Communities were dominated by Bacillota and Pseudomonadota, reflecting substrate origin, with pronounced turnover during metamorphosis and stage-specific dominance patterns, indicating developmental filtering rather than uniform microbial clearance. Crucially, dispersing larvae did not show the marked dominance signatures seen in later pupal or adult stages, supporting this stage as a pragmatic harvest window with a comparatively lower microbial-hazard indicator profile. Since downstream processing such as drying or heating will further reduce viable hazards, stage selection serves as an effective upstream control to lower the initial hazard burden entering production.

## Simple Summary

Manure-rearing insects offer a sustainable protein source for feed, but managing microbial risks is crucial. To identify an optimal harvest stage balancing yield and safety, we tracked internal bacterial communities in two fly species across development. Bacterial communities shifted significantly, with specific taxa dominating later pupal and adult stages. In contrast, dispersing-stage larvae—which stop feeding and leave the manure—maintained a more stable bacterial profile and were easier to collect. Harvesting at this dispersing stage therefore provides a practical balance between biomass production and microbial safety, supporting the design of safer manure-to-feed systems.

## 1. Introduction

Insects are the most diverse animal group, with approximately one million described species, representing 70% of all known animal species [1,2]. Several coprophagous flies—including the black soldier fly *Hermetia illucens* (L.) (Diptera: Stratiomyidae), the blow fly *Aldrichina grahami* Aldrich (Diptera: Calliphoridae), the flesh fly *Boettcherisca peregrina* Robineau-Desvoidy (Diptera: Sarcophagidae) and the house fly *Musca domestica* (L.) (Diptera: Muscidae)—can efficiently convert animal manure and food waste into larval biomass rich in protein [3,4,5]. Insect-derived protein is increasingly incorporated into animal feed formulations as a substitute for conventional protein sources such as fishmeal and soybean meal [6]. However, because insect proteins are produced via bioconversion of substrates such as swine manure, food waste and other biowastes, which can contain enteric or foodborne pathogens [7,8], the production chain must consider the possibility that flies acquire, carry, and disseminate bacteria of concern, posing potential risks to livestock and human health [9]. Importantly, regulatory acceptance of insect-derived feed ingredients and permitted rearing substrates varies among jurisdictions, and the use of manure-derived insects in the feed chain is restricted in some regions [10]. Because the regulatory status of manure-derived insects in feed varies across jurisdictions, the present study is framed as a stage-specific microbial risk-profiling analysis rather than as a recommendation for immediate feed use in any particular regulatory setting. Accordingly, the biosafety question is best framed as a risk-management problem across the production-to-processing chain rather than a binary “safe/unsafe” judgement [11].

Symbiotic microorganisms are important components of insect biology and can contribute to multiple life-history processes [12,13]. By contrast, the composition and host-associated functions of microbial communities can be dynamic, and are shaped by environmental conditions, diet, host age, and developmental stage [13,14]. In holometabolous insects, metamorphosis includes a non-feeding pupal interval and extensive tissue remodeling, processes that can restructure internal habitats and alter microbial persistence. Studies in Drosophila suggest that pupation substantially reduces larval-associated bacteria, after which adults reacquire much of their microbiota from the environment [12]. Whether similar microbiome “resets” occur in flies used or proposed for manure/biowaste bioconversion and feed-protein production, and how such dynamics affect the persistence or clearance of microorganisms of concern, including potential pathogens, remains poorly understood. This uncertainty complicates stage-specific risk assessment for manure bioconversion systems, because the developmental stage at harvest can influence the microbial load and composition entering downstream processing, even when subsequent processing is expected to reduce viable hazards [11].

While the black soldier fly (*H. illucens*) is widely regarded as the industry-standard species for organic-waste bioconversion, it is important to explore additional dipteran species that may also be suitable for particular substrates or local rearing conditions. In our preliminary screening, *A. grahami* and the flesh fly *B. peregrina* performed well on swine manure and were therefore selected as focal species in this study. Under laboratory conditions, their larvae developed rapidly, attained relatively high biomass, and efficiently utilized animal-derived substrates such as manure, supporting their potential as alternative candidates for swine-manure bioconversion [5]. However, because these flies may acquire microorganisms from manure, their internal microbial communities may include taxa of zoonotic concern, creating a potential trade-off between waste-processing utility and biosafety considerations. To address this, we characterized stage-dependent dynamics of internal bacterial communities in *A. grahami* and *B. peregrina* across multiple developmental stages spanning larval, pupal, and adult phases. Using 16S rRNA gene amplicon sequencing, we specifically addressed: (1) how internal bacterial diversity and composition change across development and (2) which developmental stage(s) may represent comparatively favorable harvest-window candidates for feed-protein production, based on cross-species patterns of internal bacterial turnover and the relative enrichment of microorganisms of concern, within a full-chain risk-assessment framework [11].

## 2. Materials and Methods

### 2.1. Insect Collection and Colony Establishment

Adult *A. grahami* and *B. peregrina* were captured in June 2024 on the campus of Nanyang Normal University (32°58′ N, 112°29′ E) using fly traps baited with swine manure. The manure was newly excreted on the day of collection, obtained from a commercial pig farm in Tanghe County, Nanyang (Henan, China), transported to the laboratory, stored at −20 °C upon arrival, and thawed before use. Species identification was based on morphological characteristics [15]. Collected specimens were transferred to separate rearing cages (35 cm × 35 cm × 35 cm) and maintained under controlled conditions: 23–25 °C, 30–40% relative humidity, and a 12:12 h L:D photoperiod. Unlimited water and approximately 50 g of bovine liver paste (replaced daily at 10:00 h) were provided. Fresh bovine liver was purchased from a local market in Nanyang (Henan, China), stored at −20 °C upon arrival, and thawed as needed for preparation of liver paste. Eggs (*A. grahami*) or larvae (*B. peregrina*, ovoviviparous) observed during liver replacement were collected to establish F1 colonies.

### 2.2. Manure Substrate Collection, Storage, and Larval Rearing

For microbial community analysis, five independent rearing replicates were established per species [16], using F1 eggs (*A. grahami*) or larvae (*B. peregrina*) reared on swine manure. Each replicate consisted of 300 larvae (hatched from eggs for *A. grahami*) reared in a plastic rearing container (9 cm i.d. × 7 cm height) containing 50 g of swine manure. The rearing containers were placed on a 5 cm layer of dry sand within a plastic chamber (30 cm × 25 cm × 15 cm) and incubated at 25 ± 0.5 °C. Swine manure was added daily at 09:00 until >50% of larvae reached the dispersing-stage (characterized by leaving the substrate to seek pupation sites). Swine manure was prepared as described above.

For manure bacterial community profiling, the substrate was homogenized and three subsamples (0.5 g each) were collected into sterile 1.5 mL microcentrifuge tubes as biological replicates. Manure subsamples (*n* = 3) were immediately frozen and stored at −80 °C until DNA extraction and sequencing and were processed in parallel with insect samples. Third-instar larvae (L3), dispersing-stage larvae (LD), pupae at 1, 3, 5, and 7 days post-pupation (P1, P3, P5, and P7; P7 only for *B. peregrina*), and newly emerged adults (A1; 1 day post-eclosion) were sampled for subsequent microbial analysis. Third-instar larvae were sampled during the late feeding phase; sampling was standardized by initiating collection when the first individuals in a cohort began to leave the manure (onset of dispersal). At that time point, L3 individuals were selected exclusively from larvae that remained within the substrate and were still feeding, whereas LD were collected only after they had actively left the substrate and had no visible manure in the gut (i.e., empty gut). Overall, we analyzed internal bacterial communities from two fly species across developmental stages (*A. grahami*: L3, LD, P1, P3, P5, A1; *B. peregrina*: L3, LD, P1, P3, P5, P7, A1) with three biological replicates per stage (*n* = 3), and manure substrate samples with three biological replicates (*n* = 3), yielding 39 insect samples and 3 manure samples (42 sequenced samples in total).

### 2.3. Sample Collection

Larvae, pupae and adults were immobilized at 4 °C and transferred to a sterile environment. Before dissection, individuals were surface-sterilized by immersion in 75% ethanol for 3 min, followed by three rinses in sterile water, following a previously published protocol [17]. This procedure was used to reduce external contamination prior to dissection; however, the final rinse water was not tested by PCR or culture-dependent methods in the present study, and residual external DNA or bacteria therefore cannot be completely excluded. For larvae and pupae, the whole-body internal contents of 10 individuals per replicate were aseptically collected, pooled, and transferred to sterile 1.5 mL microcentrifuge tubes. For adults, the entire abdominal contents of 20 individuals per replicate were aseptically dissected and pooled. Pooling was performed to ensure sufficient DNA yield for 16S rRNA sequencing and to obtain a representative community profile at the cohort level while reducing individual-to-individual variability. Accordingly, the study was designed to compare bacterial community patterns at the developmental-stage/cohort level rather than to resolve inter-individual variation within a stage. For each developmental stage, three biological replicates (*n* = 3) were prepared by selecting three of the five independent rearing replicates (one pooled sample per selected replicate). All samples were immediately frozen and stored at −80 °C until microbial analysis. Individual-level microbiomes were not profiled in this study; future work could quantify inter-individual variation within stages using single-individual sequencing.

### 2.4. DNA Extraction and Sequencing

Total microbial genomic DNA was isolated from all samples (insects and manure subsamples) employing the E.Z.N.A.^®^ Soil DNA Kit (Omega Bio-tek, Norcross, GA, USA) per manufacturer specifications. Bacterial community profiling targeted the V3–V4 hypervariable region of the 16S rRNA gene. Triplicate PCR amplifications (20 μL volume) were performed using: 4 μL 5 × TransStart FastPfu Buffer, 2 μL 2.5 mM dNTPs, 0.8 μL each of primers 338F (5′-ACTCCTACGGGAGGCAGCA-3′) and 806R (5′-GGACTACHVGGGTWTCTAAT-3′) (5 μM) [18], 0.4 μL of TransStart FastPfu DNA Polymerase, and approximately 10 ng of template DNA. Thermal cycling (ABI GeneAmp^®^9700, Applied Biosystems, Foster City, CA, USA) comprised: 95 °C for 3 min (initial denaturation); 27 cycles of 95 °C/30 s, 55 °C/30 s, 72 °C/30 s; with final extension at 72 °C for 10 min and 4 °C hold. A no-template PCR control was included during amplification to monitor contamination introduced during PCR; however, extraction blanks/kit blanks were not included during DNA extraction. Therefore, negative-control data were not available for sequencing-based contaminant identification/removal during the downstream bioinformatic workflow.

Triplicate amplicons per sample were pooled, size-fractionated via 2% agarose electrophoresis, and target bands purified (AxyPrep DNA Gel Extraction Kit, Axygen Biosciences, Union City, CA, USA). Purified products were electrophoretically verified and quantified (Quantus^TM^ Fluorometer, Promega, Madison, WI, USA). Equimolar amplicon pools were used for library construction (NEXTFLEX^®^ Rapid DNA-Seq Kit, Bioo Scientific, Austin, TX, USA). Paired-end sequencing (2 × 150 bp) was conducted on an Illumina Nextseq2000 platform (Majorbio Bio-Pharm Technology Co. Ltd., Shanghai, China). This amplicon-sequencing approach provides relative taxonomic profiles of bacterial communities rather than direct absolute quantification of bacterial load across developmental stages. The raw sequencing reads have been deposited in the NCBI Sequence Read Archive (SRA) under accession numbers SRR37603517–SRR37603549 for the insect samples and SRR28242894–SRR28242896 for the manure samples.

### 2.5. Bioinformatic Processing and Taxonomic Assignment

Raw FASTQ files were demultiplexed using custom Perl scripts. Reads were quality-filtered by fastp version 0.19.6 [19] and merged by FLASH version 1.2.7 [20] with the following criteria: (1) trimming reads at sites with average Q-score < 20 (50-bp sliding window), discarding reads < 50 bp or containing ambiguous bases; (2) merging overlapping reads with minimum overlap 10 bp and maximum mismatch 0.2; (3) assigning samples via exact barcode matching and primers (≤2 nucleotide mismatches).

High-quality sequences were clustered into operational taxonomic units (OTUs) at 97% similarity (UPARSE v11) [21]. Representative OTU sequences were taxonomically classified against the Silva v138 database (RDP Classifier v2.2 [22]; confidence threshold: 0.7).

### 2.6. Statistical Analysis

Bioinformatic analysis of the internal bacterial communities was carried out using the Majorbio Cloud platform (https://cloud.majorbio.com, accessed on 15 June 2025). Alpha-diversity indices (Chao1 and Shannon) were calculated in Mothur v1.30.2 [23] and compared among developmental stages using the Kruskal–Wallis test, followed by Dunn’s post hoc pairwise comparisons with Benjamini–Hochberg false discovery rate (FDR) correction. Beta diversity was assessed via Bray–Curtis-based PCoA (Vegan v2.5-3). Inter-group community differences were tested using ANOSIM. Differentially abundant bacterial taxa (phylum to genus) were identified via Linear discriminant analysis Effect Size (LEfSe) [24], with an LDA score > 3, *p* < 0.05.

## 3. Results

### 3.1. Sequencing Overview and Manure Substrate Community

Sequencing across all 42 samples yielded 3,231,823 high-quality merged reads in total. Of these, 3,058,394 reads were obtained from the 39 insect samples (1.31 × 10^9^ bp in total), and 173,429 reads were obtained from the 3 manure substrate samples. For the insect samples, the average read depth was 78,420 ± 2714 (mean ± SE) reads per sample (range: 50,460–126,195), with a mean merged length of 428.3 ± 0.24 (SE) bp (overall range: 200–518 bp). Rarefaction curves approached saturation for all insect samples (Figure S1). The bacterial community in the manure substrate was dominated by the phylum Bacillota (81.25% relative abundance), followed by Pseudomonadota (11.22%) and Actinomycetota (7.48%). At the genus level, *Clostridium* (26.77%), *Lactobacillus* (12.39%), *Psychrobacter* (10.52%), *Turicibacter* (10.81%), *Vagococcus* (8.88%), *Romboutsia* (8.13%), and *Corynebacterium* (6.12%) were most abundant (Figure S2).

### 3.2. Alpha Diversity

**Figure 1:**
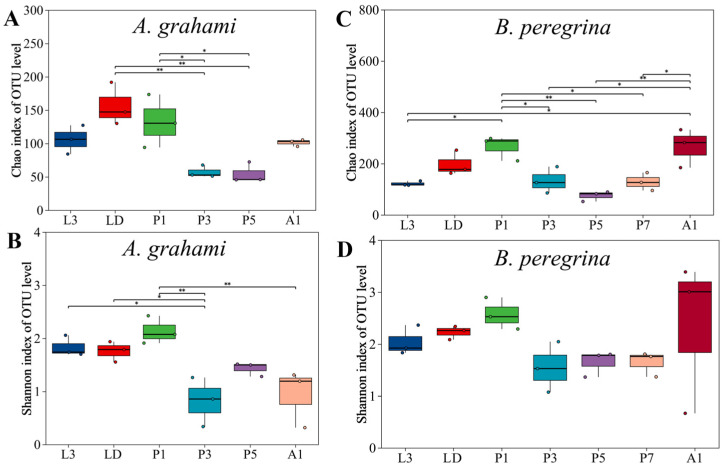
Alpha diversity of internal bacterial communities across developmental stages in *Aldrichina grahami* and *Boettcherisca peregrina*. Panels (**A**) and (**B**) show Chao1 richness and Shannon diversity, respectively, for *A. grahami*; panels (**C**) and (**D**) show Chao1 richness and Shannon diversity, respectively, for *B. peregrina*. Asterisks indicate significant pairwise differences among stages following a Kruskal–Wallis test and Dunn’s post hoc pairwise comparisons with Benjamini–Hochberg false discovery rate (FDR) correction; * indicates *p* < 0.05 and ** indicates *p* < 0.01. L3, third-instar larvae; LD, dispersing larvae; P1, P3, P5, and P7, pupae at days 1, 3, 5, and 7, respectively; A1, 1-day-old adults.

Alpha diversity showed clear stage-dependent patterns in both species (Figure 1). In *A. grahami*, alpha diversity tended to be highest from the dispersing larval stage to the early pupal stage and lowest during mid-pupal development. Specifically, Chao1 richness varied among stages (Kruskal–Wallis, *p* < 0.05; Figure 1A); richness in dispersing larvae (LD) and early pupae (P1) exceeded that in mid- and late-pupae stages (P3 and P5) (Dunn’s test, FDR-adjusted *p* < 0.05), with no other significant pairwise differences detected. Shannon diversity in *A. grahami* showed an overall pattern in which LD and P1 tended to have relatively high values, P3 showed the lowest diversity, and L3, P5, and A1 were intermediate (Figure 1B). Shannon diversity varied significantly among stages (*p* ≤ 0.01). L3, LD, and P1 did not differ significantly from one another, and each had higher Shannon diversity than P3 (FDR-adjusted *p* < 0.05); P3, P5, and A1 did not differ significantly, whereas P1 remained significantly higher than A1 (FDR-adjusted *p* < 0.05).

In *B. peregrina*, Chao1 richness was generally highest in early pupae and newly emerged adults and lowest in larvae and mid-to-late pupae, whereas Shannon diversity remained relatively stable across development. Chao1 richness varied across stages (*p* < 0.05; Figure 1C). The richness in P1 and A1 did not differ from each other, but both were significantly higher than in L3 and in the pupal stages (P3, P5, P7) (FDR-adjusted *p* < 0.05), whereas LD did not differ from any stage. Shannon diversity did not vary across stages (*p* > 0.05; Figure 1D).

### 3.3. Community Composition (Beta Diversity)

**Figure 2:**
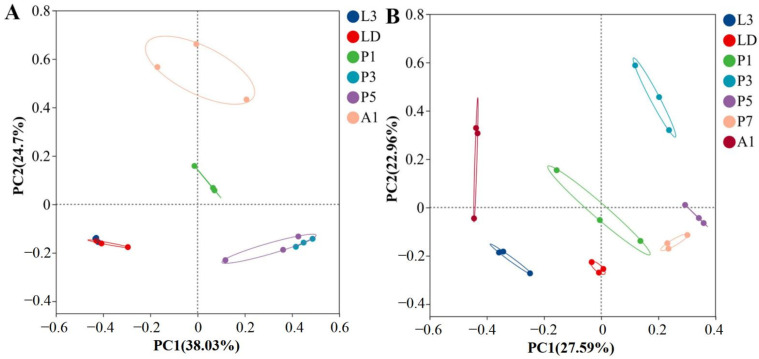
Bray–Curtis PCoA of internal bacterial community composition across developmental stages in two flies. (**A**) *Aldrichina grahami*. (**B**) *Boettcherisca peregrina*. Community composition differed significantly among stages (ANOSIM; *A. grahami*: R = 0.92, *p* ≤ 0.001; *B. peregrina*: R = 0.78, *p* ≤ 0.001). L3, third-instar larvae; LD, dispersing larvae; P1, P3, P5, and P7, pupae at days 1, 3, 5, and 7, respectively; A1, 1-day-old adults.

Principal coordinate analysis (PCoA) based on Bray–Curtis distances revealed clear separation of bacterial communities by developmental stage in both species (Figure 2). Community composition differed among stages in *A. grahami* (ANOSIM R = 0.92, *p* ≤ 0.001) and *B. peregrina* (R = 0.78, *p* ≤ 0.001). In *A. grahami*, larval samples formed a tight cluster, while pupal stages and adults (A1) occupied distinct regions in the ordination space; among the pupal samples, P3 and P5 tended to cluster more closely with each other than with P1 (Figure 2A); in *B. peregrina*, larvae separated from mid–late pupae (P3–P7), and A1 formed a distinct cluster (Figure 2B).

### 3.4. Taxonomic Shifts and Stage-Associated Taxa

**Figure 3:**
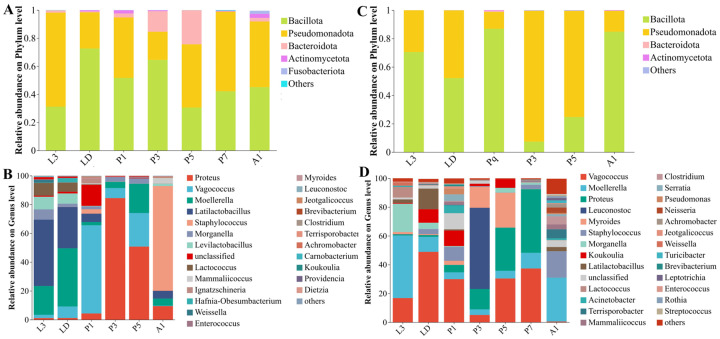
Phylum- and genus-level composition of internal bacterial communities across developmental stages in *Aldrichina grahami* and *Boettcherisca peregrina*. Relative abundances are shown at the phylum level for *A. grahami* (**A**) and *B. peregrina* (**C**), and at the genus level for *A. grahami* (**B**) and *B. peregrina* (**D**). Taxa with <1% relative abundance were grouped as “Others”. L3, third-instar larvae; LD, dispersing larvae; P1, P3, P5, and P7, pupae at days 1, 3, 5, and 7, respectively; A1, 1-day-old adults.

**Figure 4:**
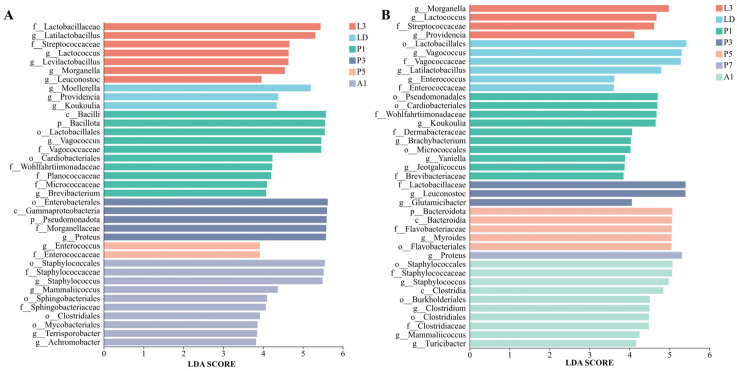
LEfSe-identified stage-discriminatory taxa (LDA score > 3) in internal bacterial communities of two flies. (**A**) *Aldrichina grahami*. (**B**) *Boettcherisca peregrina*. L3, third-instar larvae; LD, dispersing larvae; P1, P3, P5, and P7, pupae at days 1, 3, 5, and 7, respectively; A1, 1-day-old adults.

The internal bacterial communities of *A. grahami* were primarily composed of Bacillota and Pseudomonadota, and underwent a pronounced shift during pupation (Figure 3A–B). Bacillota predominated in larvae and early pupae (L3–P1) and increased again in A1, whereas Pseudomonadota increased during mid-pupation (P3–P5). At the genus level, the community composition changed markedly across development, shifting from taxa associated with Lactobacillales in larvae to *Vagococcus* in early pupae (P1), *Proteus* in mid-pupal stage, and *Staphylococcus* in adults (Figure 3B). LEfSe (LDA > 3, *p* < 0.05, Figure 4A) identified stage-discriminatory taxa consistent with these transitions, including larval Lactobacillaceae lineages, LD-associated Enterobacterales genera, P1-associated Bacillota/Lactobacillales and *Vagococcus*, P3-associated *Proteus*/Enterobacterales lineages, P5-associated *Enterococcus*, and A1-associated Staphylococcales/*Staphylococcus* (Figure 4A).

In *B. peregrina*, Bacillota and Pseudomonadota accounted for most reads across development, with Bacteroidota becoming more prominent during pupation (P3–P5) (Figure 3C). Genus-level composition showed strong stage turnover: larval stages were enriched in Enterobacterales-associated genera, LD shifted towards taxa within Lactobacillales, while pupal stages exhibited distinct dominant genera at each time point (e.g., *Leuconostoc* at P3 and *Myroides* at P5), P7 was characterized by *Proteus*, and A1 showed a reassembled community with increased Staphylococcales- and Clostridiales-associated taxa (Figure 3D). LEfSe supported these signatures (LDA > 3, *p* < 0.05), highlighting *Morganella*/*Providencia* (L3), *Vagococcus*/Lactobacillales (LD), *Leuconostoc* (P3), *Myroides* and Flavobacteriales lineages (P5), *Proteus* (P7), and *Staphylococcus* with Clostridiales-related taxa (A1) as discriminatory stage-associated signatures (Figure 4B).

## 4. Discussion

Our results show pronounced developmental turnover in the internal bacterial communities of both *A. grahami* and *B. peregrina* reared on swine manure. Community composition was strongly structured by stage (Figure 3), and richness/diversity metrics shifted in stage-dependent ways (Figure 2), indicating that progression from active feeding through dispersal, metamorphosis, and adult emergence is accompanied by major restructuring of associated bacteria. Because insects were surface-sterilized prior to dissection, and our sampling captured pooled whole-body internal contents from larvae and pupae but pooled abdominal contents from adults, the observed patterns were intended to represent internal bacterial communities in the sampled tissues while minimizing confounding from externally adherent microbes and substrate residues. Accordingly, the resulting profiles should not be interpreted as the total microbiological burden of unwashed whole insects at harvest.

To interpret these stage effects operationally, it is essential to separate “substrate signal” from host filtering. While the effects of diet and developmental stage on internal (or gut-associated) bacterial community structure are well documented in saprophagous Diptera [14,25,26], a critical and applied question for manure bioconversion systems is the relative contribution of substrate inoculation versus host-mediated filtering to the observed bacterial assemblage. The swine manure used in this experiment was dominated by Bacillota (81.25%), followed by Pseudomonadota (11.22%). Despite pronounced developmental turnover in both fly species, Bacillota and Pseudomonadota consistently remained the two most abundant phyla in *A. grahami* and *B. peregrina*. At finer taxonomic resolution, the manure microbiota was enriched for *Clostridium* (26.77%), *Lactobacillus* (12.39%), *Psychrobacter* (10.52%), *Turicibacter* (10.81%), *Vagococcus* (8.88%), *Romboutsia* (8.13%), and *Corynebacterium* (6.12%), with additional contributions from Terrisporobacter, Trichococcus, and Carnobacterium. The prominence of several manure-abundant lineages (e.g., Lactobacillales, Clostridiales) in larval stages strongly implicates substrate-derived inoculation as a primary source, consistent with prior reports [25,26]. Conversely, the stage-specific proliferation of certain taxa (e.g., Enterobacterales, *Myroides*) that were minor in manure implies potent, stage-dependent ecological filtering within the host, potentially coupled with acquisition from the rearing environment during metamorphosis. Together, these patterns support a practical “source–filter” model in which manure supplies much of the inoculum, while development determines which lineages persist, expand, or are replaced.

Our data position metamorphosis as the central ecological bottleneck within this source–filter model. In holometabolous insects, pupation entails extensive tissue remodeling and, in many taxa, substantial restructuring of internal habitats, which can impose a “bottleneck” on larval-associated microbial assemblages and promote strong community turnover across development [27,28,29]. This filtering effect is thought to be driven not only by gut histolysis and renewal, but also by stage-associated immune activation, including the production of antimicrobial peptides (AMPs), together with shedding/replacement of gut-associated structures such as the peritrophic matrix and evacuation of larval gut contents. These processes can reduce microbial carryover and help explain the partial “cleaning” of larval-associated bacteria during metamorphosis [27,30]. Consistent with this framework, pupal development in both fly species coincided with reduced richness and simplified communities at specific time points, and ordination analyses clearly separated pupae from larval stages and, in many cases, from newly emerged adults (Figure 2 and Figure 3). This pattern of ontogenetic restructuring with partial microbial carryover aligns with findings in other Diptera, reinforcing the concept that metamorphosis acts not as a complete sterilization but as a powerful selective filter [14,31,32]. Accordingly, the time-point–specific dominance patterns observed during pupation in our dataset likely reflect stage-dependent internal conditions that permit selective amplification of particular bacteria. Immediately after adult emergence, bacterial communities formed distinct clusters and showed reassembly, consistent with rapid post-eclosion restructuring under renewed exposure to environmental sources and diet, a process emphasized in Drosophila and other insects where continual reacquisition/replenishment can shape early adult microbiota [14,33]. This mechanistic context provides the basis for stage-specific biosafety reasoning in manure-based rearing systems.

Translating these ecological insights into feed production practice, the pivotal question is which developmental stage may offer a favorable balance between bioconversion utility and a comparatively lower internal microbial-hazard indicator profile. Consequently, our applied objective is not to certify any stage as safe feed, but to identify species-stage combinations associated with a comparatively lower internal microbial-hazard indicator profile under our conditions. Risk-profile frameworks for insects used as food/feed emphasize that biological hazards are shaped by substrate, rearing hygiene, and processing, and therefore should be evaluated along the full chain from production to the final product [11]. Within that framework, our internal-community data nominate LD as a pragmatic harvest-window candidate for further evaluation, rather than establishing it as the safest stage. At LD, larvae have ceased feeding and leave the substrate, which may reduce direct manure carryover during collection; correspondingly, LD did not coincide with the pronounced stage-specific internal dominance patterns that emerged later in development (Figure 4). In *A. grahami*, LD was characterized by Enterobacterales-associated taxa (LEfSe), whereas the most conspicuous dominance signatures occurred during pupation and early adulthood (e.g., *Proteus* at P3, *Enterococcus* at P5, and *Staphylococcus* at A1; Figure 4A). In *B. peregrina*, LD was dominated by Lactobacillales-associated taxa (notably *Vagococcus*; LEfSe), while distinct pupal or adult peaks were observed at later time points (e.g., *Leuconostoc* at P3, *Myroides* at P5, *Proteus* at P7, and adult reassembly at A1; Figure 4B). However, because the present analyses were conducted on surface-sterilized insects, this inference pertains to internal bacterial profiles only and does not capture surface-associated microbes or manure residues that may also contribute to harvest-stage biosafety. Thus, LD should be viewed as a priority stage for follow-up validation, not as a definitive safety endpoint. Adults add an additional biosafety dimension because filth flies can disperse and mechanically disseminate microorganisms acquired from microbe-rich substrates [34,35]. Downstream processing steps commonly used for insect meals—particularly drying and/or heat treatment—are expected to reduce viable microbial hazards in the final product; however, because the present study focused on internal bacterial communities of surface-sterilized insects, stage selection should be interpreted only as a potential upstream measure for reducing the initial internal hazard indicator profile entering processing, rather than as evidence of final product safety [11].

It is critical to acknowledge the limitations of 16S rRNA gene amplicon data in this biosafety context. These data provide taxonomic profiles rather than functional virulence assessments, reflect relative abundance rather than absolute bacterial load, and cannot distinguish commensal from virulent strains within the same genus. Thus, taxa that appear relatively enriched at a given developmental stage may still occur at lower absolute abundance than in earlier feeding stages, particularly if total bacterial load declines during metamorphosis. In addition, because microbiome analyses were based on pooled, surface-sterilized samples, the present study was limited to stage-level internal bacterial community patterns rather than inter-individual variation or the total microbiological burden of unwashed insects, including surface-associated microbes. The frozen–thawed manure substrate used here may also not fully represent the microbial composition or the full high-risk pathogen profile of fresh manure under industrial rearing conditions, and reagent-derived contamination—particularly in low-biomass samples such as pupae—cannot be completely excluded because extraction blanks were not available. Moreover, the modest sample size for microbiome analysis (*n* = 3 biological replicates per developmental stage) may not fully capture the variability of manure-reared populations and warrants cautious interpretation of LEfSe-derived stage-associated taxa as exploratory, hypothesis-generating signatures rather than definitive biomarkers. Future work should incorporate absolute quantification, targeted pathogen/ARG assays, assessment of surface-associated microbiota, and validation of downstream processing efficacy [31]. Accordingly, our results should be interpreted as a comparative, hypothesis-generating hazard ranking under the present rearing conditions rather than as direct evidence that any developmental stage is safe for animal feed use. Even with these caveats, the consistent stage structuring across both flies and the practical advantage of collecting larvae after gut evacuation justify prioritizing LD in future validation studies as a candidate stage with a comparatively lower internal microbial-hazard indicator profile in swine-manure bioconversion systems.

## 5. Conclusions

Our data support a coherent source–filter narrative with practical implications. Internal bacterial communities in *A. grahami* and *B. peregrina* shift markedly across development, reflecting both manure-derived inputs and stage-specific filtering. These dynamics are relevant to biosafety reasoning: metamorphosis was associated with strong community restructuring, and adults may represent an additional dissemination concern. Under our conditions, dispersing-stage larvae (LD) emerged as a pragmatic candidate harvest stage with a comparatively lower internal microbial-hazard indicator profile. This conclusion should be interpreted as hypothesis-generating rather than as direct evidence of feed safety, and will require further validation through pathogen-targeted assays, assessment of surface-associated microbiota, and evaluation of downstream processing efficacy.

## Data Availability

The Raw data for the 16S rRNA gene presented in this study are openly available in NCBI Sequence Read Archive (SRA) database at https://www.ncbi.nlm.nih.gov/, accession numbers: SRR37603517–SRR37603549 for the insect samples and SRR28242894–SRR28242896 for the manure samples.

## Supplementary Materials

The following supporting information can be downloaded at: https://www.mdpi.com/article/10.3390/insects17040353/s1, Figure S1: Shannon rarefaction curves of internal bacterial communities in two flies. (A) *Aldrichina grahami* (B) *Boettcherisca peregrina*; Figure S2: Taxonomic composition of the swine-manure substrate used for larval rearing. Relative abundance at the phylum level (A) and genus level (B); taxa with <1% relative abundance were grouped as “Others”.
