# Supplementary material for: Internal Microbiota Guided Stage Selection in Two Swine-Manure Bioconversion Flies for Feed-Protein Harvest
Source: Insects. 2026 Mar 24;17(4):353. doi: 10.3390/insects17040353 (PMC13116670; doi:10.3390/insects17040353)
Supplement: Supplementary file 1 [file insects-17-00353-s001.zip › insects-4155591-supplementary.pdf]

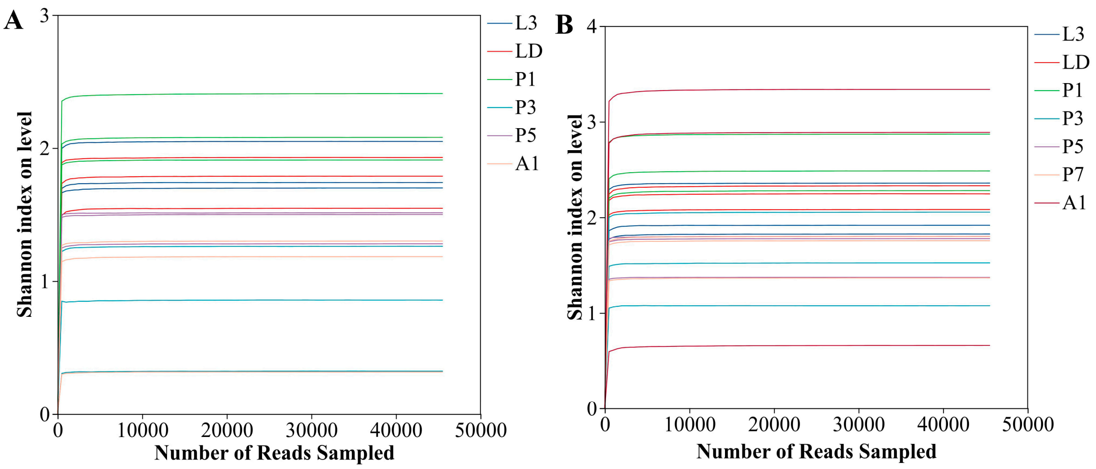

Fig. S1 Shannon rarefaction curves of internal bacterial communities in two flies. (A) *Aldrichina grahami*. (B) *Boettcherisca peregrina*.

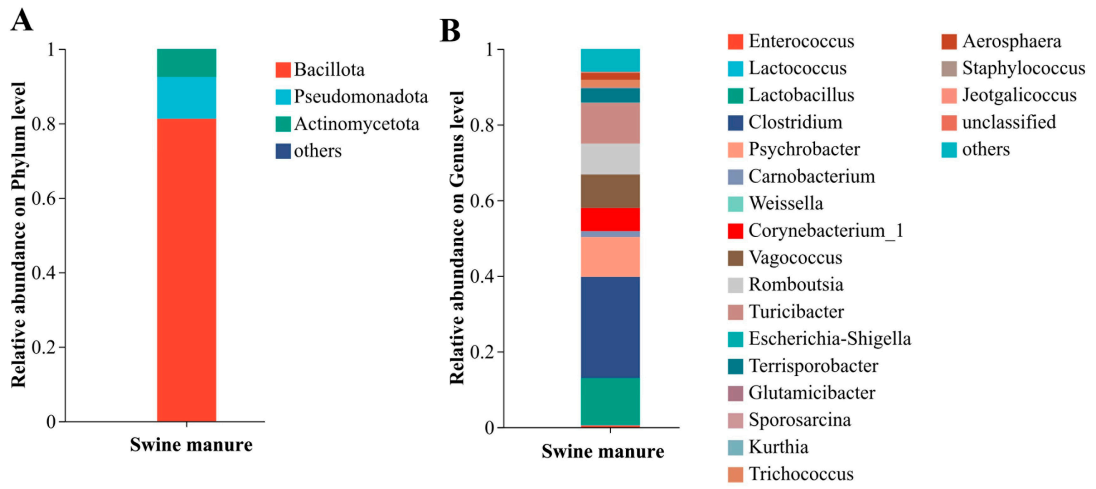

Fig. S2. Taxonomic composition of the swine-manure substrate used for larval rearing. Relative abundance at the phylum level (A) and genus level (B); taxa with <1% relative abundance were grouped as “Others”.
